# Supplementary material for: MetaRibo-Seq measures translation in microbiomes
Source: Nat Commun. 2020 Jun 29;11:3268. doi: 10.1038/s41467-020-17081-z (PMC7324362; doi:10.1038/s41467-020-17081-z)
Supplement: Supplementary file 10 — Supplementary Data 7 [file 41467_2020_17081_MOESM10_ESM.zip › File2/Confidence_High_Taxonomy/425529_out.krona.html]

Javascript must be enabled to view this page.

members
magnitude
magnitudeUnassigned
count
unassigned
taxon
rank

425529\_out

320

superkingdom
320
2

976
320
phylum

class
200643
320

320
171549
order

319
815
family

genus
6

SRS014683\_contig\_number\_3215SRS016989\_contig\_number\_12431SRS022524\_contig\_number\_contig-100\_413.86432SRS053398\_contig\_number\_contig-100\_131.143679SRS057478\_contig\_number\_549SRS140513\_contig\_number\_11059
319
816


SRS045739\_contig\_number\_1116
1
291644
species


SRS055966\_contig\_number\_263
817
1
species

28116
2

SRS020622\_contig\_number\_1081SRS022713\_contig\_number\_281
species


SRS1041134\_contig\_number\_12243
291645
1
species

species
1
371601

SRS023971\_contig\_number\_4717

species
47678
3

SRS014948\_contig\_number\_15886SRS019030\_contig\_number\_12322SRS048164\_contig\_number\_13522


SRS017433\_contig\_number\_441SRS017859\_contig\_number\_3390SRS049402\_contig\_number\_7026SRS049773\_contig\_number\_16979SRS050520\_contig\_number\_1222SRS075984\_contig\_number\_25647SRS105082\_contig\_number\_1178SRS1054716\_contig\_number\_9561SRS1055069\_contig\_number\_1354SRS1055076\_contig\_number\_10184SRS140492\_contig\_number\_11153SRS142542\_contig\_number\_12949SRS142890\_contig\_number\_28604SRS143722\_contig\_number\_4168SRS147377\_contig\_number\_8975SRS148817\_contig\_number\_10571SRS893288\_contig\_number\_641
17
46506
species

species

SRS045826\_contig\_number\_14627SRS147653\_contig\_number\_8117SRS150029\_contig\_number\_21031
357276
3


SRS014459\_contig\_number\_4098SRS893172\_contig\_number\_2659
2
246787
species

species

SRS011084\_contig\_number\_11809SRS011134\_contig\_number\_29894SRS011239\_contig\_number\_11156SRS011302\_contig\_number\_24909SRS011405\_contig\_number\_9049SRS012849\_contig\_number\_22041SRS012902\_contig\_number\_229SRS013158\_contig\_number\_776SRS013382\_contig\_number\_266SRS013521\_contig\_number\_8522SRS013638\_contig\_number\_18820SRS013687\_contig\_number\_14975SRS013965\_contig\_number\_41838SRS014313\_contig\_number\_4581SRS014412\_contig\_number\_4534SRS014534\_contig\_number\_2025SRS014736\_contig\_number\_2231SRS014855\_contig\_number\_14695SRS014979\_contig\_number\_10570SRS015065\_contig\_number\_31488SRS015190\_contig\_number\_1721SRS015217\_contig\_number\_1132SRS015369\_contig\_number\_6086SRS015486\_contig\_number\_433SRS015578\_contig\_number\_27397SRS015579\_contig\_number\_15430SRS015663\_contig\_number\_35755SRS015694\_contig\_number\_2944SRS015782\_contig\_number\_25764SRS015854\_contig\_number\_973SRS016056\_contig\_number\_2895SRS016095\_contig\_number\_12564SRS016132\_contig\_number\_404SRS016203\_contig\_number\_11628SRS016335\_contig\_number\_25341SRS016495\_contig\_number\_16397SRS016629\_contig\_number\_2928SRS016753\_contig\_number\_3300SRS016840\_contig\_number\_1561SRS016897\_contig\_number\_186SRS016954\_contig\_number\_9542SRS017247\_contig\_number\_3034SRS017307\_contig\_number\_2633SRS017375\_contig\_number\_214SRS017521\_contig\_number\_15690SRS017701\_contig\_number\_10474SRS017745\_contig\_number\_595SRS018351\_contig\_number\_15435SRS018427\_contig\_number\_14783SRS018623\_contig\_number\_13966SRS018817\_contig\_number\_2361SRS018836\_contig\_number\_20731SRS018888\_contig\_number\_4392SRS018936\_contig\_number\_10037SRS019068\_contig\_number\_19444SRS019178\_contig\_number\_2425SRS019267\_contig\_number\_7712SRS019381\_contig\_number\_8712SRS019397\_contig\_number\_9890SRS019445\_contig\_number\_25740SRS019496\_contig\_number\_3181SRS019601\_contig\_number\_29201SRS019693\_contig\_number\_9121SRS019808\_contig\_number\_20830SRS019910\_contig\_number\_6679SRS019968\_contig\_number\_10058SRS020328\_contig\_number\_27861SRS020508\_contig\_number\_4SRS020869\_contig\_number\_24666SRS021219\_contig\_number\_714SRS021484\_contig\_number\_17027SRS021948\_contig\_number\_14922SRS023526\_contig\_number\_22454SRS023583\_contig\_number\_3269SRS023829\_contig\_number\_46SRS023914\_contig\_number\_5727SRS024009\_contig\_number\_2984SRS024075\_contig\_number\_1620SRS024331\_contig\_number\_27685SRS024492\_contig\_number\_12946SRS024625\_contig\_number\_10257SRS042284\_contig\_number\_1764SRS042966\_contig\_number\_15118SRS043411\_contig\_number\_1455SRS043667\_contig\_number\_5785SRS044535\_contig\_number\_24498SRS045004\_contig\_number\_2067SRS045244\_contig\_number\_2247SRS045645\_contig\_number\_519SRS046068\_contig\_number\_4411SRS046369\_contig\_number\_4901SRS046502\_contig\_number\_14042SRS046810\_contig\_number\_contig-100\_15.16SRS047014\_contig\_number\_21060SRS047044\_contig\_number\_6119SRS047433\_contig\_number\_11123SRS049446\_contig\_number\_1760SRS049712\_contig\_number\_22295SRS049896\_contig\_number\_28756SRS049959\_contig\_number\_16600SRS049995\_contig\_number\_13520SRS050026\_contig\_number\_596SRS050422\_contig\_number\_14088SRS050752\_contig\_number\_19387SRS050801\_contig\_number\_3740SRS050941\_contig\_number\_13522SRS050974\_contig\_number\_1807SRS050998\_contig\_number\_1868SRS051031\_contig\_number\_3788SRS051610\_contig\_number\_9729SRS051882\_contig\_number\_3017SRS052027\_contig\_number\_16962SRS052576\_contig\_number\_66SRS052697\_contig\_number\_20545SRS053087\_contig\_number\_2714SRS053214\_contig\_number\_5472SRS053335\_contig\_number\_14858SRS054059\_contig\_number\_contig-100\_32.48735SRS054352\_contig\_number\_19SRS054905\_contig\_number\_17142SRS055017\_contig\_number\_30679SRS056537\_contig\_number\_12678SRS057480\_contig\_number\_10013SRS058268\_contig\_number\_48SRS058723\_contig\_number\_1632SRS062427\_contig\_number\_9983SRS062654\_contig\_number\_5750SRS062701\_contig\_number\_1861SRS063127\_contig\_number\_1075SRS063190\_contig\_number\_10299SRS063370\_contig\_number\_3850SRS063489\_contig\_number\_4181SRS063518\_contig\_number\_5654SRS063813\_contig\_number\_5930SRS063928\_contig\_number\_451SRS064557\_contig\_number\_748SRS064645\_contig\_number\_2242SRS064973\_contig\_number\_108SRS065504\_contig\_number\_34489SRS074586\_contig\_number\_contig-100\_0.58413SRS074670\_contig\_number\_4437SRS074964\_contig\_number\_1948SRS075078\_contig\_number\_11893SRS075398\_contig\_number\_1066SRS075716\_contig\_number\_2550SRS075773\_contig\_number\_24722SRS075821\_contig\_number\_18723SRS076756\_contig\_number\_16916SRS076876\_contig\_number\_1064SRS076976\_contig\_number\_19958SRS077194\_contig\_number\_3897SRS077231\_contig\_number\_16597SRS077294\_contig\_number\_12410SRS077454\_contig\_number\_15343SRS077502\_contig\_number\_14039SRS077552\_contig\_number\_6302SRS077641\_contig\_number\_23612SRS077730\_contig\_number\_918SRS077849\_contig\_number\_22007SRS077991\_contig\_number\_contig-100\_54.23961SRS078176\_contig\_number\_11455SRS078242\_contig\_number\_6000SRS078665\_contig\_number\_9311SRS097889\_contig\_number\_14623SRS097920\_contig\_number\_20822SRS097958\_contig\_number\_3004SRS098061\_contig\_number\_1244SRS098073\_contig\_number\_17658SRS098571\_contig\_number\_29616SRS098644\_contig\_number\_27265SRS098827\_contig\_number\_206SRS098881\_contig\_number\_454SRS100021\_contig\_number\_314SRS100907\_contig\_number\_8721SRS103987\_contig\_number\_2104SRS104036\_contig\_number\_6844SRS104084\_contig\_number\_7734SRS1041031\_contig\_number\_26374SRS1041032\_contig\_number\_17188SRS1041036\_contig\_number\_710SRS1041037\_contig\_number\_818SRS1041039\_contig\_number\_296SRS1041090\_contig\_number\_5649SRS1041091\_contig\_number\_9711SRS1041092\_contig\_number\_3073SRS1041095\_contig\_number\_2560SRS1041116\_contig\_number\_8002SRS1041118\_contig\_number\_13828SRS1041129\_contig\_number\_14043SRS1041130\_contig\_number\_1975SRS1041133\_contig\_number\_14139SRS1041136\_contig\_number\_24817SRS1041138\_contig\_number\_2835SRS1041139\_contig\_number\_315SRS1041140\_contig\_number\_20885SRS1041141\_contig\_number\_8925SRS1041142\_contig\_number\_1174SRS1041143\_contig\_number\_6114SRS1041144\_contig\_number\_3906SRS1041147\_contig\_number\_769SRS1041157\_contig\_number\_3980SRS1041164\_contig\_number\_307SRS104327\_contig\_number\_3041SRS104400\_contig\_number\_12995SRS104485\_contig\_number\_8SRS104693\_contig\_number\_10547SRS104912\_contig\_number\_14608SRS104975\_contig\_number\_2682SRS1054928\_contig\_number\_1125SRS1055022\_contig\_number\_11937SRS1055038\_contig\_number\_852SRS1055049\_contig\_number\_10508SRS1055056\_contig\_number\_12830SRS1055083\_contig\_number\_522SRS1055095\_contig\_number\_842SRS142503\_contig\_number\_8961SRS142599\_contig\_number\_16823SRS142781\_contig\_number\_111SRS142923\_contig\_number\_13586SRS142980\_contig\_number\_3063SRS143070\_contig\_number\_12261SRS143085\_contig\_number\_1506SRS143148\_contig\_number\_19641SRS143342\_contig\_number\_12111SRS143372\_contig\_number\_241SRS143417\_contig\_number\_18149SRS143876\_contig\_number\_11642SRS143895\_contig\_number\_17175SRS143991\_contig\_number\_1722SRS144183\_contig\_number\_42501SRS144297\_contig\_number\_9095SRS144362\_contig\_number\_1150SRS144506\_contig\_number\_37978SRS144537\_contig\_number\_20201SRS144603\_contig\_number\_12167SRS144714\_contig\_number\_contig-100\_337.168289SRS144753\_contig\_number\_12248SRS145308\_contig\_number\_1072SRS146764\_contig\_number\_16733SRS146813\_contig\_number\_16886SRS147022\_contig\_number\_136SRS147039\_contig\_number\_3343SRS147425\_contig\_number\_1665SRS147766\_contig\_number\_3047SRS147977\_contig\_number\_10144SRS148196\_contig\_number\_36113SRS148253\_contig\_number\_14163SRS148319\_contig\_number\_2038SRS148424\_contig\_number\_2443SRS148784\_contig\_number\_21007SRS148970\_contig\_number\_348SRS149075\_contig\_number\_3108SRS149244\_contig\_number\_2399SRS149325\_contig\_number\_14537SRS892882\_contig\_number\_16069SRS893170\_contig\_number\_6652SRS893187\_contig\_number\_1394SRS893252\_contig\_number\_1248SRS893253\_contig\_number\_242SRS893256\_contig\_number\_299SRS893259\_contig\_number\_1405SRS893270\_contig\_number\_20510SRS893279\_contig\_number\_14537SRS893285\_contig\_number\_730SRS893300\_contig\_number\_11568SRS893327\_contig\_number\_1016SRS893341\_contig\_number\_1024SRS893358\_contig\_number\_2941SRS893366\_contig\_number\_10027SRS893369\_contig\_number\_9619SRS893373\_contig\_number\_14914SRS893378\_contig\_number\_10110SRS893380\_contig\_number\_305SRS893382\_contig\_number\_178SRS893383\_contig\_number\_9216SRS971275\_contig\_number\_30315SRS971276\_contig\_number\_17921
821
277

329854
1

SRS014287\_contig\_number\_10101
species


SRS012969\_contig\_number\_11074
1
2292944
species

species

SRS147271\_contig\_number\_37057
310297
1

species
1
29523

SRS077589\_contig\_number\_3692


SRS147614\_contig\_number\_2611
1
820
species

family
1
2005525

375288
1
genus


SRS015431\_contig\_number\_21991
1
1263091
species
